# Histone deacetylase (HDAC) 9: versatile biological functions and emerging roles in human cancer

**DOI:** 10.1007/s13402-021-00626-9

**Published:** 2021-07-27

**Authors:** Chun Yang, Stéphane Croteau, Pierre Hardy

**Affiliations:** 1 Research Center of CHU Sainte-Justine, University of Montréal, 3175 Côte-Sainte-Catherine, Room 2.17.004, Montréal, Québec H3T 1C5 Canada; 2 Departments of Medicine, Pediatrics, Pharmacology and Physiology, University of Montréal, Montréal, QC Canada

**Keywords:** Histone deacetylase (HDAC) 9, Myocyte enhancer-binding factor 2-interacting transcriptional repressor (MITR), Tumorigenesis, Cancer development, Signaling pathways

## Abstract

**Background:**

HDAC9 (histone deacetylase 9) belongs to the class IIa family of histone deacetylases. This enzyme can shuttle freely between the nucleus and cytoplasm and promotes tissue-specific transcriptional regulation by interacting with histone and non-histone substrates. HDAC9 plays an essential role in diverse physiological processes including cardiac muscle development, bone formation, adipocyte differentiation and innate immunity. HDAC9 inhibition or activation is therefore a promising avenue for therapeutic intervention in several diseases. HDAC9 overexpression is also common in cancer cells, where HDAC9 alters the expression and activity of numerous relevant proteins involved in carcinogenesis.

**Conclusions:**

This review summarizes the most recent discoveries regarding HDAC9 as a crucial regulator of specific physiological systems and, more importantly, highlights the diverse spectrum of HDAC9-mediated posttranslational modifications and their contributions to cancer pathogenesis. HDAC9 is a potential novel therapeutic target, and the restoration of aberrant expression patterns observed among HDAC9 target genes and their related signaling pathways may provide opportunities to the design of novel anticancer therapeutic strategies.

## A brief introduction to histone deacetylases

Epigenetic modifications play a vital role in gene regulation because they can alter DNA and histone structures [1]. Histone deacetylases (HDACs) catalyze the removal of acetyl groups from lysine residues at the amino termini of histones. Because this action causes chromatin to condense into a transcriptionally repressed conformation, HDACs are mainly involved in gene silencing [2–4]. Some studies have recently identified additional non-histone HDAC targets, including several cytoplasmic and nuclear proteins. HDACs interact with both histone and non-histone targets to regulate a variety of cellular processes including proliferation, differentiation, migration and cell death [5, 6]. Emerging evidence suggests that altered HDAC activity can also promote carcinogenesis in human subjects by regulating the acetylation of critical oncogenic and tumor suppressor proteins [3].

The eighteen currently identified mammalian HDACs belong to one of four different classes based on their structural similarity, enzymatic function and intracellular location. Class I HDACs (HDACs 1, 2, 3 and 8) are expressed ubiquitously. These proteins contain a HDAC catalytic domain and are localized within the cell nucleus. In contrast, class II HDACs (HDACs 4, 5, 6, 7, 9 and 10) are expressed more selectively and can undergo bi-directional translocation between the nucleus and cytoplasm. Class III HDACs (sirtuins [SIRTs] 1–7) encompass seven proteins that are structurally and functionally distinct from all other HDACs [7]. Lastly, the class IV HDAC (HDAC 11) localizes mainly in the nucleus. The class I, II and IV HDACs are zinc-dependent amidohydrolases, whereas the catalytic activity of class III HDACs is dependent on nicotinamide adenine dinucleotide (NAD^+^). Verdin et al. [8] presented a phylogenic tree documenting the links between all known human HDACs.

## HDAC9 is a class IIa HDAC

The class II HDACs are further sub-divided into class IIa (HDACs 4, 5, 7 and 9) and class IIb (HDACs 6 and 10). Class IIa HDACs contain a highly conserved C-terminal catalytic domain with a very weak deacetylase activity, and they share a N-terminal regulatory domain that facilitates their interactions with transcription factors. In contrast, class IIb HDACs contain duplicated HDAC domains, which distinguishes them from class IIa HDACs [9]. Verdin et al. [8] presented schematic structures for each class II HDAC. The class IIa HDACs are large (120–135  kDa) relative to other zinc-dependent HDACs, and they maintain a hallmark capacity to undergo nucleo-cytoplasmic transport. All class II HDACs include a nuclear export signal (NES) and a nuclear localization signal (NLS), which permit them to shuttle between the nucleus and the cytoplasm. Asfaha et al. [10] provided a comprehensive review of the detailed structural features of class IIa HDACs and the regulation of their activities. Nucleo-cytoplasmic transport of class IIa HDACs primarily depends on the phosphorylation state of the enzyme. The specific kinases and phosphatases that regulate nucleo-cytoplasmic localization of class IIa HDACs have been reviewed by Parra and Verdin [11]. Likewise, DiGiorgio and Brancolini [12] and Mathias et al. [13] recently reviewed the role of other posttranslational modifications, including ubiquitination, sumoylation, acetylation and proteolytic cleavage, in the regulation of class IIa HDAC activity.

Class IIa HDACs repress gene transcription in a variety of tissues. Their activity primarily depends on tissue-specific gene expression, the recruitment of critical cofactors and nucleo-cytoplasmic shuttling [14]. They additionally depend on the formation of multiprotein complexes at their respective C-termini. One such complex, for example, contains HDAC3, the silencing mediator for retinoid and thyroid receptors that is also known as the nuclear receptor co-repressor (N-CoR), and the mammalian SIN3 transcription regulator family member A (SIN3A) [15–17]. In contrast, the N-terminal domain mainly supports protein-protein interactions and post-translational modifications [8, 18].

**Fig. 1:**
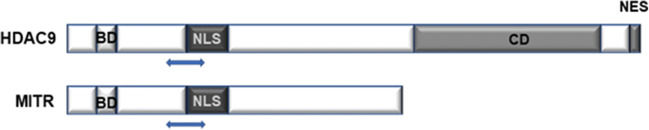
Schematic diagram of functional domains of HDAC9 and MITR. HDAC9 contains a MEF2 binding domain (BD) at the N-terminus, a nuclear localization signal (NLS), a C-terminal catalytic domain (CD) and a nuclear export signal (NES) near the C-terminus. MITR is a splice variant of HDAC9 that lacks a HDAC CD domain and NES. Double arrow heads indicate the region that HDAC9 and MITR share (450-600 amino acids) in their N-terminal domains with other members of class IIa HDACs

The gene for human HDAC9 is located on chromosome 7p21.1 and encodes multiple protein isoforms. The full-length HDAC9 protein consists of 1,069 amino acids encoded by a sequence within exons 2–26 (exon 1 is untranslated) [15]. An ortholog of *Xenopus* myocyte enhancer-binding factor 2-interacting transcriptional repressor (MITR), also known as histone deacetylase-related protein (HDRP), is an example of a well-characterized truncated splice isoform of HDAC9. Human MITR is 590 amino acids in length and has no catalytic domain [15]. It consists primarily of the noncatalytic N-terminal region of HDAC9, which includes 450–600 amino acids that are conserved among several of the class IIa HDACs (i.e., HDACs 4, 5 and 7; Fig.  1). Human MITR is fully capable of transcriptional repression even though it lacks the catalytic domain, which suggests that the HDAC9 catalytic domain (and likely the catalytic domain of all class IIa HDACs) is not necessarily required for transcriptional repression [19]. A smaller isoform, HDAC9a, has a 132-amino acid deletion at the C-terminus [20].

Petrie et al. [15] cloned several alternatively spliced variants of HDAC9 from the COS-7 monkey fibroblast-like cell line. The HDAC9Δ7 variant contains only 1,025 amino acids, as result of a deletion of exon 7. HDAC9Δ12 is 981 amino acids in length due to an in-frame deletion of exon 12, HDAC9 Δ7/Δ12 has deletions of both exon 7 and exon 12, and HDAC9Δ15 is 1,027 amino acids in length and lacks the sequence encoded by exon 15. Although the biological consequences of each of these splicing variants remain unclear, several studies have suggested that each isoform may have unique biological properties based on their distinct cellular localization patterns and expression levels in various tissues [20].

## Specific HDAC9 expression patterns

Transcripts encoding HDAC9 are expressed at various levels in many normal human tissues and cell lines (Table 1) and have been detected in the brain, skeletal muscle, colon, thymus, spleen, kidney, placenta, lung, bone marrow, fetal brain and fetal liver. HDAC9 is highly expressed in brain and skeletal muscles, but has only been detected at low levels or, in some cases, not detected at all in the heart [15, 20]. The HDAC9 homolog MITR is expressed at comparable levels in human heart, brain and skeletal muscle and at very low levels in placenta, lung, liver, kidney and pancreas [21]. Within the hematopoietic system, both HDAC9 and MITR are preferentially expressed in lymphoid and monocytic cells [15]. HDAC9 is also highly expressed in the mouse brain, including the hippocampus, cerebral cortex, basolateral amygdaloid nuclei and choroid plexus. Although it is exclusively expressed in post-mitotic and mature neurons, HDAC9 has not been detected in adult neural stem cells, glia cells, astrocytes or oligodendrocytes [22].

**Table 1:** Relative expression of HDAC9 and MITR in tissues/organs and cell lines

	HDAC9 expression [references]	MITR expression [references]
Tissues/organs		
Brain	++ [15, 20]	++ [15, 21]
Skeletal muscle	++ [15, 20]	++ [15, 19, 21]
Heart	+ [20] ± [15]	+ [15, 19, 21]
Liver	± [20]	+ [15, 21]
Bone marrow	+ [15]	+ [15]
Spleen	+ [15]	+ [15, 19]
Thymus	+ [15]	+ [15]
Pancreas	+ [20]	+ [21]
Placenta	+ [15, 20]	+ [15, 21]
Kidney	+ [15, 20]	+ [15, 21]
Lung	+ [15, 20]	+ [19, 21]; ± [15]
Colon	+ [15]	+ [15]
Fetal brain	++ [15]	++ [15]
Fetal liver	+ [15]	+ [15]
* Xenopus* stomach, intestine, gall bladder		+ [19]
* Xenopus* stomach mouse tailbud embryo		+ [19]
Adult mouse brain: post-mitotic and mature neurons	+ [22]	
Adult mouse neural stem cells, glia, astrocytes, oligodendrocytes	– [22]	
Mouse embryogenic myocardial chambers and interventricular septum	+ [23, 24]	+ [23, 24]
Cell lines		
plasma cell line: U266	+ [15]	
CD14^+^ monocyte/macrophages	++ [15]	
CD19^+^ B cells	+ [15]	
Monkey COS-7 cell line		+ [21]
Mouse myoblast C2C12 cell line		+ [21]

Note: ++, high expression; +, moderate expression; ±, very low or absent; –, undetectable

Sparrow et al. [19] explored MITR expression in developing embryos and adult tissues of several frogs from the genus *Xenopus*. They found that MITR was expressed in mature somites at the neurula stage of early embryos, but in later stages of development MITR was only expressed in muscle tissue. In adult tissues, MITR was detected at comparatively low levels in the intestine, stomach, gall bladder, spleen, skeletal, heart and lung [19].

MITR expression patterns in mouse tissues are significantly different from those in *Xenopus*. Specifically, MITR has been detected in mouse heart, brain and skeletal muscle during embryogenesis and at high levels in the heart, brain and spleen of adult mice, whereas it is expressed at very low levels in the lungs, liver, skeletal muscles and kidneys of adult mice [23]. MITR expression has also been detected in monkey COS-7 cells [21].

## Regulation of HDAC9 activity and its role in modulating transcription

HDAC9 expression and activity are tightly regulated at the transcriptional, post-transcriptional and post-translational levels. The mechanisms involved in the transcriptional regulation of HADC9 have been studied in a variety of different tissues and conditions. For example, transcription of the *HDAC9* gene has been found to be upregulated during cardiac and skeletal muscle differentiation [25]. Myocyte enhancer factors of the MEF2 family have been characterized as important transcriptional regulators of all class IIa HDACs. MEF2A, MEF2C and MEF2D can all bind to the proximal promoter region of *HDAC9* [12, 25]. HDAC9 is specifically upregulated by the oncogenic protein Ras in senescent human fibroblasts [26]. In addition, nuclear factor kappa-light-chain-enhancer of activated B-cells (NF-κB), early growth response protein 1 (Egr-1) and paired-box containing 5 (Pax-5) can all induce HDAC9 expression [12].

Several recent studies have found that microRNAs (miRs) are involved in post-transcriptional downregulation of HDAC9. Specifically, HDAC9 has been found to be downregulated by miR-188 in bone marrow stromal cells [27], by miR-361-5p in cells associated with cardiac hypertrophy [28], by miR-936 or miR-101-3p in retinoblastoma cells [29, 30], by miR-383-5p in gastric carcinoma cells [31], by miR-211-5p in bladder cancer cells [32], by miR-30d-5p in esophageal squamous cell carcinoma cells [33], by miR-509 3p in non-small cell lung cancer cells [34] and by miR-377 in oral squamous cell carcinoma cells [35].

Post-translational phosphorylation may also modulate the activity of HDAC9 by controlling its subcellular localization [12]. In their recent review, Brancolini et al. [36] presented a simplified diagram showing the main domains of HDAC9 that contain phosphorylation sites. The subcellular localization of HDAC9 is controlled by the phosphorylation of at least three serine residues in HDAC9 (Ser220, Ser451 and Ser611) that facilitate binding interactions with dimers of 14-3-3 molecular chaperone proteins [14]. Binding interactions between HDAC9 and 14-3-3 proteins can mask the NLS to prevent nuclear import of HDAC9 and/or unmask the NES to promote direct interactions between chromosomal region maintenance protein 1 (CRM1, also known as exportin1) and nuclear export [37]. For example, heterologous expression of salt inducible kinase (SIK) isoforms SIK2 or SIK3 results in a dramatic re-localization of cytosplasmic HDAC9 via phosphorylation of HDAC9 at conserved motifs and stimulation of 14-3-3 binding [38]. HDAC9 can also be phosphorylated at Ser242 by the mitotic kinase Aurora B. This phosphorylation event facilitates the re-localization of HDAC9 at the mitotic midzone during late anaphase and within the midbody during cytokinesis [39].

Several recent studies have suggested that phosphorylation within the NLS, along with chaperone 14-3-3 binding, may also promote the redistribution of MITR. Harrison et al. [40] demonstrated that phosphorylation of Ser253 affects the nuclear exclusion of MITR via the action of calcium calmodulin-dependent protein kinase (CaMK). CaMK-catalyzed phosphorylation of MITR at Ser218 and Ser448 in COS cells facilitates a 14-3-3-mediated blockade of the MITR/MEF2 interaction [23]. Moreover, phosphorylation of Ser243 within the NLS of MITR results in its cytoplasmic retention, which is accompanied by decreases in MEF2-mediated transcriptional suppression in C2C12 myoblasts [41].

Sumoylation is another reversible post-translational modification in which a small ubiquitin-like modifier (SUMO) is covalently attached to a target protein. Sumoylation modifies subcellular localization, protein stability and protein-protein interactions [42]. MITR is a particularly potent substrate for sumoylation, as indicated by modifications detected on nearly all MITR proteins modified *in vitro* using a SUMO-based modification system [43].

Like other class IIa HDACs, HDAC9 does not bind directly to DNA. It instead interacts with several partners through distinct domains and is then recruited to the promoters of its target genes via its interactions with DNA sequence-specific transcription factors (for an excellent review on the interaction partners of class IIa HDACs, see reference [8]). HDAC9 interacts and co-localizes *in vivo* with several transcriptional repressors and co-repressors, including the oncogenic protein TEL and N-CoR [15]. Although it has no intrinsic HDAC enzymatic activity, MITR forms complexes with other HDACs (including HDAC 1, 3, 4, 5 and others) in HEK293T human embryonic kidney cells [44]. MITR also interacts with MEF2 and represses MEF2-mediated transcription via (1) direct binding of MITR to HDAC1 [19], (2) sumoylation of the C-terminal transcriptional activation domain of MEF2, which is promoted by MITR transcription [45] and (3) interactions between MITR and the transcriptional co-repressor C-terminal-binding protein [44]. HDAC9 not only targets histones, i.e., recent studies have reported HDAC9-mediated reversible acetylation of a large group of nonhistone proteins including transcription factors, receptors, signal transducers and intracellular chaperones. The following sections describe these newly identified targets and functional partners of HDAC9 based on their physiologic roles and potential contributions to cancer development.

## Physiological roles and targets of HDAC9

**Fig. 2:**
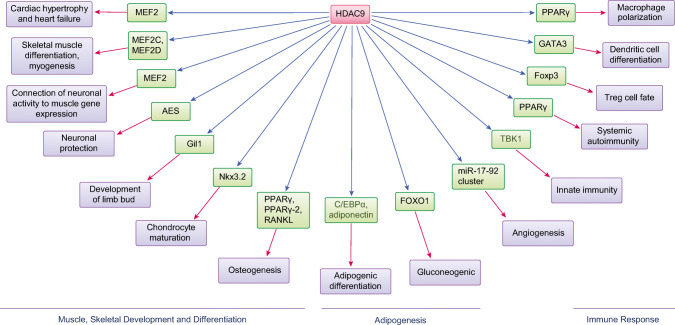
The major physiological roles of HDAC9

A large body of research has demonstrated that HDAC9 regulates a wide variety of physiological responses and many targets. Studies of HDAC9 KO (knockout) and transgenic (TG) mice suggest a crucial role for HDAC9 in myocyte and adipocyte differentiation as well as the development of cardiac muscle [24, 25, 46]. HDAC9 has also been implicated in providing support for immune and metabolic processes and for protection of the nervous system, among other activities. HDAC9-mediated immune and metabolic dysregulations have been linked to pathological processes associated with human diseases, as recently reviewed by Hu et al. [36] and Brancolini et al. [36, 47]. Below, we will focus on our current understanding of the function of HDAC9 and its role(s) in several major important physiological processes (summarized in Table 2; Fig. 2).

**Table 2:** Major physiological roles of HDAC9

Role	Upstream regulators	Downstream targets and signaling	References
Regulates cardiac development and controls cardiac hypertrophy	Long non-coding RNAs MEG3 and miR-361-5p	Hypertrophic signaling and MEF2	[24, 46]
Negatively regulates muscle differentiation	MEF2	MEF2	[25]
Connects neuronal activity to gene expression in muscle tissue		AChR, MEF2	[48]
Maintains the neuronal-based functions of the brain and prevents neuronal death		AES	[22, 49, 50]
Regulates development of the limb bud		Gli1and Shh signaling	[51]
Controls chondrocyte viability and hypertrophic maturation		Nkx3.2, and the PIASy-RNF4 axis	[52]
Suppresses osteoclast differentiation, promotes osteogenesis, and inhibits adipogenesis of BMSCs	miR-188	PPARγ/RANKL signaling, PPARγ-2, and FABP4	[27, 53–55]
Regulates adipogenic differentiation		C/EBPα abd adiponectin	[56–58]
Regulates gluconeogenesis		FOXO1	[59–61]
Regulates macrophage polarization		ABCA1, ABCG1, and PPARγ	[62]
Activates antiviral innate immunity	Dnmt3a	TBK1-IRF3 signaling and type I IFNs	[63]
Regulates effector T cell-mediated systemic autoimmunity		PPARγ	[64]
Controls regulatory T cell function		Foxp3 and STAT5	[65–67]
Regulates DC differentiation		GATA3	[68]
Promotes angiogenesis		miR-17-92 cluster	[69, 70]

### HDAC9 suppresses the fetal gene program and hypertrophic growth in the adult heart

HDAC9 is expressed in developing myocardial chambers and the interventricular septum during embryogenesis [23, 24], which suggests that HDAC9 plays a role in cardiac development. Although HDAC9 KO mice exhibit no noticeable anatomical anomalies (note that no functional HDAC9 or MITR proteins can be detected in homozygous mutants), brain and neurological deficits have been reported. HDAC9 KO mice develop cardiac hypertrophy as they age and in response to cardiac pressure overload. They are also hypersensitive to calcineurin-mediated hypertrophy [46]. Cardiac hypertrophy is closely associated with increased morbidity and mortality in various cardiovascular disorders [71]. MITR can effectively repress fetal gene expression and is the predominant form of HDAC9 expressed in the heart. Mechanistically, MITR blocks signals that promote cardiac hypertrophy by downregulating the acetylation of fetal gene promoters. Calcineurin-medicated super-activation of MEF2 activity in HDAC9 KO mice also suggests that MEF2 is a downstream target of HDAC9 *in vivo.* These findings identify HDAC9 and MITR as critical modulators of hypertrophic signaling pathways [46]. Zhang et al. [28] investigated the role of the long non-coding RNA (lncRNA) MEG3 (maternally expressed gene 3) in the progression of cardiac hypertrophy. They found that MEG3 contributes to the pathogenesis of cardiac hypertrophy by competing with miR-361-5p binding and, consequently, upregulating HDAC9 expression. MEG3 may, therefore, function as a positive regulator in the setting of cardiac hypertrophy via its interaction with the miR-361-5p/HDAC9 axis.

### HDAC9 is a critical negative regulator of muscle differentiation

MEF2 factors, most notably MEF2C and MEF2D, are highly expressed in skeletal and cardiac muscle in a pattern similar to that of HDAC9 [23, 72, 73]. Haberland et al. [25] found that MEF2 is a potent activator of the *HDAC9* promoter and can directly upregulate the expression of HDAC9 in skeletal muscle. HDAC9 interacts with MEF2 proteins and suppresses their transcriptional activity by establishing corepressor complexes. HDAC9, therefore, serves as a negative regulator of the muscle gene expression program.

### HDAC9 integrates neuronal activity with acetylation of muscle chromatin and gene expression

In skeletal muscle, HDAC9 may repress activity-dependent genes after birth, as increased expression of specific genes has been observed following denervation of HDAC9 KO mice. For example, Mejat et al. [48] found that HDAC9 was downregulated in response to denervation, resulting in increased chromatin acetylation and expression of the acetylcholine receptor (AchR). MITR interacts with both HDAC1 and HDAC3 to attenuate denervation-induced histone H3 hyperacetylation and suppression of MEF2 transcriptional activity in murine skeletal muscle. Forced MITR expression in denervated muscle prevents chromatin acetylation and repression of activity-dependent genes. MITR may, therefore, contribute to the control of activity-dependent regulation of skeletal muscle genes by inducing histone H3 deacetylation at the promoter of its target genes via its interaction with MEF2 [48].

### HDAC9 plays a protective role in the nervous system

In the adult nervous system, HDAC9 is expressed only in post-mitotic and mature neurons, which suggests that HDAC9 may play a vital role in maintaining neuronal function in the mature brain. Studies focused on the neuropathology of schizophrenia have specifically implicated the neuronal protective role of HDAC9. For example, hemizygous deletions of HDAC9 have been identified in a small fraction of schizophrenia patients [22]. Furthermore, reduced expression of both HDAC9 and MITR in cells derived from the central nervous system results in neuronal cell death [49]. Zhang et al. [50] demonstrated that AES (amino enhancer of split, a member of the Groucho family of transcriptional repressors) promotes neuronal apoptosis and that MITR is involved in direct interactions that permit it to block AES-induced neuronal cell death.

### HDAC9 regulates the development of the limb bud

Mice devoid of HDAC9 exhibit post-axial polydactyly and present with an extra hallux on the right hind foot [51]. The morphogenic signaling protein Shh (sonic hedgehog) is a critical regulator of digit formation and increased Shh-mediated signaling in the developing limb bud results in polydactyly [74]. The transcription factor Gli1 (glioma-associated oncogene homolog 1) is a downstream mediator of Shh signaling and is highly expressed in tissues from the feet of perinatal HDAC9 KO mice. Morrison et al. [51] reported that MITR functions as a negative upstream regulator of Gli1 in the Shh-mediated signaling cascade. The absence of HDAC9 may, therefore, result in hyper-activation of the Shh pathway and thus explain the polydactyly observed in these mice.

### HDAC9 controls hypertrophic maturation of chondrocytes

Nkx3.2 (NK3 homeobox 2, also known as Bapx1) is a homeodomain-containing transcription factor that inhibits hypertrophic maturation of chondrocytes during chondrogenesis [75, 76]. Choi et al. [52] showed that HDAC9-induced deacetylation of Nkx3.2 plays a critical role in controlling its stability. HDAC9-dependent deacetylation of Nkx3.2 specifically triggers PIASy (protein inhibitor of activated STAT)-mediated sumoylation and subsequent ring finger protein 4 (RNF4)-mediated ubiquitination. RNF4 is a poly-SUMO-specific E3 ubiquitin ligase. Post-translational modification of Nkx3.2 by HDAC9 plays a crucial role in controlling chondrocyte maturation during skeletal development in vertebrates.

### HDAC9 modulates bone development and formation

Skeletal maintenance relies on both osteoclast-mediated bone resorption and concomitant bone formation (also known as osteogenesis) [77]. RANKL (receptor activator of the NF-κB ligand) and the nuclear receptor transcription factor PPARγ (peroxisome proliferator-activated receptor-gamma) play critical roles in osteoclast differentiation and osteoclastogenesis [78]. HDAC9 KO mice exhibit elevated rates of bone resorption and low bone density. At the molecular level, HDAC9 forms a negative regulatory loop that modulates both PPARγ and RANKL signaling to promote suppression of osteoclast differentiation and bone resorption. These results suggest that HDAC9 can function as a physiological modulator of bone remodeling and skeletal homeostasis [53].

Li et al. [27] further demonstrated that miR-188 regulates the differentiation of BMMSCs (bone marrow mesenchymal stem cells) during aging and contributes to age-related bone loss. Interestingly, HDAC9 is a direct target of miR-188, i.e., miR-188 inhibits HDAC9 expression via post-transcriptional modifications and upregulates PPARγ expression during differentiation of BMMSCs into adipocytes [27]. Chen et al. [54] similarly indicated that MITR can serve as a switch that promotes the formation of bone and inhibits adipogenesis from BMMSCs by inactivating PPARγ-2. MITR is highly expressed in osteoblasts and bone tissues and minimally expressed in adipocytes. MITR accelerates BMMSC-derived osteogenesis and attenuates MSC-derived adipogenesis via its interaction with PPARγ-2 and by suppressing the expression of the adipocyte marker fatty acid-binding protein 4 (FABP4).

Chen et al. [54] also explored associations between MITR expression and the potential of human and mouse primary BMMSCs to differentiate into osteoblasts and/or adipocytes at different ages. They found that MITR was highly expressed in osteoblasts differentiated from BMMSCs isolated from young individuals and that higher levels of MITR expression were associated with stronger induction of osteogenic differentiation and weaker induction of adipogenic differentiation in BMMSCs from younger individuals. These studies collectively suggest that MITR critically modulates both osteogenesis and adipogenesis from BMMSCs in an age-dependent manner [55].

### HDAC9 negatively regulates adipocyte differentiation

HDAC9 is more highly expressed in visceral than in subcutaneous preadipocytes. Chatterjee et al. [56] found that HDAC9 negatively regulates adipogenic differentiation via a deacetylase-independent mechanism. Preadipocytes of HDAC9 KO mice exhibit rapid adipogenic differentiation, whereas overexpression of HDAC9 in 3T3-L1 preadipocytes suppresses their differentiation. In preadipocytes, HDAC9 is recruited together with USF1 (upstream stimulatory factor 1) to the promoter region of the CCAAT/enhancer-binding protein-α (C/EBPα) gene. USF1 regulates the expression of several genes that contribute to both glucose and lipid metabolic pathways and is the key enzyme mediating the lipogenic response to fasting and insulin signaling [79]. C/EBPα is a USF1-regulated gene that serves as a master regulator of adipocyte differentiation [80]. Downregulation of HDAC9 during adipocyte differentiation leads to its dissociation from the USF1 complex. This results in increased expression of C/EBPα. HDAC9 is also involved in the formation of adipose tissue and metabolic dysfunction associated with a high-fat diet (HFD) [57]. Upregulated HDAC9 blocks adipogenic differentiation in mice maintained chronically on a HFD, which results in the accumulation of improperly differentiated adipocytes and a reduced expression of adiponectin, a key regulator of glucose and lipid metabolism. Results from an exome array meta-analysis performed by Spracklen et al. [58] support the involvement of HDAC9 in the regulation of circulating adiponectin levels.

### HDAC9 regulates gluconeogenesis

HDAC9 is selectively expressed in insulin-producing β-cells in both the embryonic and adult pancreas. Lenoir et al. [59] reported that HDAC9-deficient mice developed increased β-cell mass, which suggests that HDAC9 expression may have an inhibitory effect on the development of pancreatic β-cells. Chen et al. [60] identified HDAC9 as a mediator of hepatic gluconeogenesis and glucose homeostasis. Upregulated HDAC9 deacetylates forkhead box protein O 1 (FOXO1) and consequently enhances gluconeogenesis in the livers of patients with hepatitis C virus (HCV) and transgenic mice with persistent HCV. FOXO1 is an important gluconeogenic transcription factor that mediates the effects of insulin on hepatic metabolism [81]. HDAC9-mediated deacetylation of FOXO1 promotes the expression of genes associated with gluconeogenesis, including phosphoenolpyruvate carboxykinase and glucose-6-phosphatase. It additionally promotes the expression of the gluconeogenic transcription factors PPARγ coactivator-1α, cyclic AMP-responsive element-binding protein and the glucocorticoid receptor [61].

### HDAC9 regulates macrophage polarization

HDAC9 is highly expressed during macrophage differentiation. Macrophages lacking HDAC9 express high levels of ABCA1 (ATP-binding cassette transporter A1) and ABCG1 (ATP-binding cassette transporter G1) and can, therefore, suppress foam cell formation by accelerating cholesterol efflux. HDAC9 deletion further causes macrophages to switch from the pro-inflammatory M1 to the anti-inflammatory M2 phenotype via the actions of PPARγ. At the molecular level, HDAC9 deficiency results in the accumulation of acetylated histone H3 at ABCA1, ABCG1 and PPARγ promoters in macrophages. These results broadly suggest that HDAC9 plays an important role in the regulation of macrophage function [62].

### HDAC9 activates antiviral innate immunity

TANK-binding kinase 1 (TBK1) affects the activation of the innate immune antiviral response. TBK1 is specifically required for activating interferon regulatory factor 3 and inducing type I interferons (e.g., IFN-α and IFN-β) [82]. Zhang et al. [63] demonstrated that HDAC9 participates in direct interactions with TBK1 via its deacetylase domain, resulting in enhanced TBK1 kinase activity and an overall increase in the production of type I IFNs. DNA methyltransferase 3A (Dnmt3a) is highly expressed in peritoneal macrophages and selectively upregulates the expression of IFN-α and IFN-β. In response to innate immune stimuli, Dnmt3a in naïve peritoneal macrophages upregulates the expression of HDAC9. HDAC9 then catalyzes the deacetylation of lysine residues in TBK1 so that the cells can respond by fully activating the TBK1-IRF3 signaling pathway and producing type I IFNs to promote host defense [63].

### HDAC9 acts as an epigenetic switch in effector T cell-mediated systemic autoimmunity

Yan et al. [64] described a unique and tissue-specific role for HDAC9 in promoting CD4^+^ T cell plasticity, inflammation and autoimmunity. HDAC9 deficiency in autoimmune MRL/lpr (Murphy Roths large/lymphoproliferation) mice that have abnormal effector T cell function leads to (1) decreased immunoproliferation, inflammation, proteinuria and autoantibody production, and improved survival, (2) a reduced autoimmune phenotype secondary to decreased expression of follicular and extrafollicular T cell genes and augmented Th2 polarization and (3) downregulation of cytokines and chemokines secondary to increased PPARγ promoter activity.

### HDAC9 controls the fate of regulatory T cells

Forkhead box P3 (Foxp3) is involved in several characteristic immune responses. Thymic-derived Foxp3^+^ regulatory T cells (Tregs) are essential for sustaining immune homeostasis [65]. HDAC9 is highly expressed in Foxp3^+^ Tregs and is particularly important for the critical regulation of Foxp3-dependent suppression. Upon T cell receptor-mediated stimulation, HDAC9 is phosphorylated and exported from the nucleus, thereby facilitating the acetylation of several Foxp3 lysine residues. This sequence of events is critical for the function of the Treg suppressor [66].

Beier et al. [67] showed that HDAC9-mediated deacetylation of signal transducer and activator of transcription 5 (STAT5) regulates its stability and activity. STAT5 plays a critical role in supporting Foxp3^+^ Treg function by modulating the interleukin 2-mediated signaling pathway [83]. De Zoeten et al. [65] also noted the importance of HDAC9-mediated regulation of host inflammatory responses in a study on the role of HDAC9 in disease pathogenesis in a mouse colitis model. In that model, colitis was associated with increased local expression of HDAC9, and HDAC9 KO mice were resistant to the development of pathology.

### HDAC9 regulates the differentiation of CD8^+^ dendritic cells (DCs)

CD8^+^ DCs play important roles in the generation of cytotoxic T lymphocytes that are specific for cell-associated antigens [84]. The number of CD8^+^ cells in human lung cancer stroma samples positively correlates with the expression of HDAC9. Ning et al. [68] reported that HDAC9-deficient DCs show upregulated expression of the transcription factor GATA binding protein 3 (GATA3), which promotes T-lymphocyte development. HDAC9 expression in DCs may play a critical role in regulating the transcription of lymphoid-related gene programs that promote differentiation of CD8^+^ DCs.

### HDAC9 promotes angiogenesis

Kaluza et al. [85] performed both *in vitro* and *in vivo* experiments revealing a strong pro-angiogenic effect of HDAC9. HDAC9 KO mice exhibit reduced retinal vessel outgrowth and impaired recovery of blood flow after a period of hindlimb ischemia. HDAC9 has been shown to repress the transcription of miR-17-92 *in vitro*. MiR-17-92 is a polycistronic miR cluster that encodes miR-17, miR-18a, miR-19a/b, miR-20a and miR-92a, which are known for their anti-angiogenic activity. The pro-angiogenic effects of HDAC9 may, therefore, be mediated by HDAC9-mediated repression of the anti-angiogenic miR-17-92 cluster [69]. A subsequent study found that HDAC9 was strongly expressed in pancreatic ductal adenocarcinoma, especially in tissues with an abundant vasculature. This finding suggests a possible pro-angiogenic role of HDAC9 in tumor angiogenesis [70].

The aforementioned studies collectively illustrate the diverse physiological roles played by HDAC9. Because of the important role of HDAC9 in cell development and differentiation, dysregulated HDAC9 expression and activity have been associated with several human diseases including systemic autoimmunity, diabetes, the development of atherosclerotic plaques and cardiovascular disease [64, 86–89]. Hu et al. [47] recently reviewed the role of HDAC9 in the pathogenesis of diabetes and other chronic diseases. At the molecular level, HDAC9 may induce histone deacetylation to inhibit target gene transcription. HDAC9 may alternatively interact directly with and/or deacetylate non-histone proteins to alter their functions. Several known upstream regulators and downstream targets of HDAC9 and its functional partners are presented in Table 2. These findings collectively suggest that restoration of aberrant expression patterns observed among HDAC9 target genes and their related signaling pathways may provide novel therapeutic options.

## HDAC9 expression, regulation and activity in cancer

Emerging data suggest that there are several potential roles for HDAC9 in tumorigenesis. For example, upregulated HDAC9 expression is a characteristic of many different neoplastic tissues [90], and HDAC9 interacts *in vivo* with numerous transcriptional repressors and oncogenic proteins that have been implicated in tumorigenesis [15]. HDAC9 may also be able to regulate anti-tumor immune responses by decreasing CD8^+^ DC and T cell infiltration into the tumor microenvironment [68]. The contributions of HDAC9 to specific types of cancer have been studied worldwide. Current information linking aberrant expression of HDAC9 to various tumors and related transcription factors is summarized in Table 3.

**Table 3:** Roles of HDAC9, its upstream regulators and its downstream targets in human cancers

Cancer type	Cells and tissues	Roles	Upstream regulators	Downstream targets	Ref.
Glioblastoma (GBM)	Glioma tissues and primary glioma cells, and human GBM cell lines U87 and LN229	Promotes GBM cell growth and tumor formation		TAZ	[91]
Medulloblastoma (MB)	Primary tumor tissues of MB patients	Increases rate of cell growth and viability			[92]
Breast cancer (BC)	Serum from recurrent BC and TNBC patients and TNBC cell lines MDA-MB-231, MDA-MB-1739, and HCC1395	Promotes invasion and tumor angiogenesis		miR-206	[93, 94]
	Tumor tissues from BC patients and human BC cell lines MCF-7 and BT474	Promotes BC cell proliferation, migration, and invasion			[95]
	Aggressive BC cell lines, including luminal cell lines ZR75, T47D, SKBR3, BT474, and HCC1500, and basal cell lines HCC1937, SUM149, MDA231, MDA436, Hs578T, BT549, and HBL100	Promotes proliferation and decreased apoptosis of target cells		SOX9	[96]
	Paclitaxel-resistant TNBC MDA-MB-231 and tumor tissues from BC patients	Increases paclitaxel resistance in TNBCs		IL11	[97]
	ERα-negative tumor tissues from a BC patient and ERα-negative BC cell lines MDA‐MB231 and MDA‐MB436	Increases antiestrogen resistance in BC cells		ERα	[98]
Hepatocellular carcinoma (HCC)	Tumor tissues from HCC patients, differentiated HCC cell lines HepG2 and HuH1, HCC cell lines Hep3B, Huh7, and PLC, undifferentiated HCC cell lines HLE and HLF, and OXA-resistant HCC cells	Increases cell growth, reduces apoptosis, and increases anchorage-independent cell growth	miR-376a	miR-376a, ALDH1A3	[99–103]
Pancreatic ductal adenocarcinoma (PDAC)	PDAC tissues and cell lines CFPAC-1, HPAC, SW1990, and Capan-2	Promotes PDAC cell proliferation and migration			[104]
Oral squamous cell carcinoma (OSCC)	Tumor tissues from OSCC patients and UPCI-SCC-116 cells	Promotes cell growth, and inhibits apoptosis	miR-377	MEF2D, NR4A1	[35, 105]
Cutaneous squamous cell carcinoma (CSCC)	Tumor tissues from CSCC patients	Tumorigenesis			[106]
Leiomyosarcoma (LMS)	Tumor tissues from LMS patients and LMS cell lines SK-UT-1, SK-LMS-1, MES-SA, and DMR	Sustains proliferation and survival of LMS cells		FAS	[107–109]
Osteosarcoma	Tumor tissues from osteosarcoma patients and osteosarcoma cell lines U2OS and MG63	Promotes cell proliferation and invasion		p53	[110]
Childhood acute lymphoblastic leukemia (ALL)	Bone marrow samples from children diagnosed with ALL				[111, 112]
Acute myeloid leukemia (AML)	AML cell line HL60 induced with sodium valproate				[112, 113].
B-cell non-Hodgkin lymphoma (B-NHL)	B-NHL cell lines and primary samples from B-NHL patients	Promotes cell growth and survival		BCL6, p53	[15, 114, 115]
Gastric cancer (GC)	Tumor tissues from GC patients and human GC cell lines SGC-7901, BGC-823, MKN-45, AGS, MGC-803, and HGC-27	Promotes cell proliferation and reduces apoptosis	miR-383-5p		[31, 116]
Lung cancer; non-small cell lung cancer (NSCLC)	Tumor tissues from NSCLC patients and NSCLC cell lines SK-MES-1, H522, H460, and A549	Promotes cell proliferation, reduces cell apoptosis, and enhances the progression of NSCLC	CBR3-AS1, miR 509 3p		[34, 117, 118]
Esophageal squamous cell carcinoma (ESCC)	Tumor tissues from ESCC patients and esophageal carcinoma TE1 cells	Promotes cell proliferation, migration, invasion, amd enhances the epithelial-mesenchymal transition process	miR-30d-5p		[33]
Bladder cancer (BCa)	BCa cell lines 5637 and T24 and urine from patients diagnosed with urothelial BCa	Promotes cell proliferation, migration, and invasion, and reduces cell apoptosis	miR-211-5p		[32, 119]
Retinoblastoma (Rb)	Tumor tissues from Rb patients and human Rb cell lines Y79 and WERI-Rb-1	Promotes cell proliferation and reduces apoptosis	miR-936, miR-101-3p		[29, 30, 120]
BRM-negative carcinoma	BRM-negative cell lines SW13 and C33A	Sustains growth of carcinoma cell		BRM	[121]

### Glioblastoma (GBM)

GBM (grade IV glioma) is the most common and most lethal primary brain tumor [122]. Yang et al.[91] analyzed HDAC9 expression in a large cohort of GBM patients and found high levels in 472 out of 504 glioma cases and that 33 of the 88 patient cases in the TCGA (The Cancer Genome Atlas) dataset were associated with a poor prognosis. HDAC9 is commonly expressed in human GBM cell lines (e.g., U87 and LN229) and in primary cultured patient-derived GBM cells. HDAC9 is essential for GBM cell growth *in vitro* and promotes the formation of U87 tumors in immunodeficient mice. HDAC9 interacts with a transcriptional co-activator with a PDZ-binding motif (TAZ) to enhance its expression in GBM cells. TAZ is an oncogene that enhances the activity of the epidermal growth factor receptor (EGFR), which is a crucial signaling molecule that accelerates cell proliferation and promotes tumorigenesis [123]. Current results, therefore, suggest that HDAC9 may activate the TAZ-mediated EGFR signaling pathway to promote GBM development.

### Medulloblastoma (MB)

MB is the most common primary intracranial malignant tumor in pediatric patients and accounts for ~ 20 % of all pediatric brain tumors. MB is invasive and fast-growing and patients diagnosed with MB typically have poor clinical outcomes [124]. Milde et al. [92] performed mRNA expression profiling of primary MB samples and found that HDAC9 was highly expressed in subgroups with poor prognoses compared to those with low-risk MB. They also found that high levels of HDAC9 were associated with lower rates of survival. Gene expression arrays and immunohistochemical staining of MB tissue sections revealed immunoreactive HDAC9 protein in most primary MB samples that was predominantly localized in the cytoplasm. HDAC9 knockdown studies in established MB cell lines (e.g., Daoy, UW228-2, UW228-3, ONS76 and Med8A) led to significant decreases in the rates of cell growth and viability. These results suggest that HDAC9 expression may be a valuable marker for risk stratification and prognosis in patients diagnosed with MB.

### Breast cancer (BC)

Bera et al. [93] found significantly higher HDAC9 levels in serum samples from patients with recurrent triple-negative BC (TNBC) than in patients with nonrecurrent BC. TNBC cells do not express estrogen receptors (ERs), progesterone receptors or human epidermal growth factor receptor 2 (HER2). TNBC is also one of the most aggressive forms of BC and is typically associated with a poor prognosis, so HDAC9 expression may serve as a significant biomarker to trace recurrent TNBC. Higher levels of HDAC9 expression were also found in TNBC tissues compared to tissues from patients with non-TNBC. HDAC9 inhibition decreases the invasiveness of TNBC cells and effectively blocks tumor angiogenesis *in vivo* [94]. Interestingly, miR-206 has been found to be downregulated in both TNBC cell lines and TNBC tumor tissues. miR-206 suppresses the expression of vascular endothelial growth factor (VEGF), mitogen-activated protein kinase 3 (MAPK3) and SRY-box transcription factor 9 (SOX9), thereby inhibiting TNBC cell invasion and angiogenesis [125]. Inhibition of HDAC9 in TNBC cells results in increased expression of miR-206 accompanied by decreased expression of VEGF and MAPK3. HDAC9 may, therefore, contribute to TNBC invasiveness and tumor angiogenesis by suppressing miR-206 expression [94]. Moreover, upregulated HDAC9 expression was also detected in human BC cell lines, including MCF-7 and BT474. One study additionally found that high levels of HDAC9 were associated with poor prognoses in Chinese female BC patients [95]. This study reported that HDAC9 expression levels were correlated with lymph node metastasis and TNM stage. *In vitro* experiments revealed that downregulation of HDAC9 in BC cells inhibits cell proliferation, migration and invasion [95].

HDAC9 is not only overexpressed in the most aggressive human BC cell lines, but is also strikingly overexpressed in basal cells (e.g., HCC1937, SUM149, MDA231, MDA436, Hs578T, BT549 and HBL100) relative to luminal cells [96]. Lapierre et al. [96] analyzed a cDNA array dataset containing mRNA profiles from 184 BC patients together with findings listed in a public dataset (GSE2250). Their results further confirmed increased HDAC9 expression in basal tumor cells. HDAC9 expression was also correlated with SOX9 expression and was associated with a poor prognosis. Exogenous expression of HDAC9 in MCF-7 cells increased their proliferation and decreased their rate of apoptosis in association with dysregulated expression of cell cycle and apoptosis regulators, including cyclin-dependent kinase inhibitor 1A, BAX and TNF receptor superfamily member 10a.

The microtubule inhibitor paclitaxel is currently the most widely used drug for the treatment of TNBC [126]. Lian et al. [97] reported MITR enrichment in paclitaxel-resistant cells and suggested that MITR might be a critical modulator of paclitaxel resistance in TNBC MDA-MB-231 cells. Mechanistically, MITR interacts with and represses the actions of MEF2A, thereby resulting in transcriptional inhibition of interleukin (IL) 11 and, thereby, activation of the downstream JAK/STAT3 signaling pathway. JAK/STAT signaling plays a critical role in BC tumorigenesis, maintenance and metastasis [127]. MITR may, therefore, be a promising predictive biomarker of paclitaxel responsiveness in TNBC tumors.

Estrogens also play crucial roles in the pathogenesis of BC, and anti-estrogen-based endocrine therapies are commonly used to treat ER-positive BC. HDAC9 is strongly overexpressed in anti-estrogen-resistant MCF-7 BC cells and ERα-negative BC cell lines, including MDA‐MB231 and MDA‐MB436. Expression of HDAC9 is associated with decreased ERα expression and reduced ERα-mediated transcriptional activity in MCF-7 cells, and HDAC9-overexpressing BC cells are less sensitive to anti-estrogens. Moreover, high levels of HDAC9 expression have been found to be positively associated with gene upregulation and to be correlated with a poorer prognosis in endocrine therapy-resistant BC relative to BC patients who respond to antiestrogen therapy. It is possible that interaction between HDAC9 and ERα signaling in BC cells contributes to hormone therapy resistance [98].

### Hepatocellular carcinoma (HCC)

HCC is the most frequently diagnosed aggressive primary liver cancer and is a leading cause of cancer-related death worldwide. Several independent studies have indicated that HDAC9 may promote the development of HCC and may also serve as a potential prognostic indicator of the disease. In one such study, Freese et al. [99] systematically analyzed the expression of different HDAC classes in HCC cells and tissues and found increased HDAC9 expression in HCC tissues compared to tumor-free liver. An additional analysis of the TCGA dataset revealed that high expression of HDAC9 was significantly correlated with a poor patient survival.

Hu et al. [100] similarly evaluated HDAC9 expression in tumors and para-cancerous tissues from 37 patients diagnosed with HCC. Higher levels of HDAC9 mRNA were found in the HCC tissues, and HCC patients with higher HDAC9 expression levels had poorer prognoses. Zheng et al. [128] reported overexpression of HDAC9 in 41 HCC specimens at levels that were inversely correlated with miR-376a, and further demonstrated that HDAC9 directly downregulates miR-376a expression in human HCC Huh7 cells via a mechanism involving site-specific deacetylation of H3K18 in its upstream region. Interestingly, miR-376a directly downregulates HDAC9 expression in this cell line, and overexpression of miR-376a inhibits HCC cell proliferation and promotes apoptosis [101]. Because miR-376a is frequently downregulated in HCC cell lines and primary tissues, autoregulation of HDAC9 may be an important factor in the development of HCC via a miR-376a/HDAC9 regulatory circuit [101].

Kanki et al. [102] recently found that HDAC9 is preferentially expressed in undifferentiated HCC, including HLE and HLF cells. Results from the analysis of a TCGA dataset and *in vitro* experiments suggest that HDAC9 may regulate differentiation and the acquisition of stemness in HCC cells. HDAC9 is also involved in maintaining anchorage-independent growth via aldehyde dehydrogenase 1A3 (ALDH1A3) regulation, which is a stemness-related gene. Clinically, oxaliplatin (OXA)-based systemic chemotherapy has been effective for treating advanced HCC, although resistance limits its survival benefit in HCC patients [129]. Liang et al. [103] reported that HDAC9 is highly expressed in HCC cells in association with OXA resistance, suggesting that HDAC9 may play an important regulatory role in this setting.

### Pancreatic ductal adenocarcinoma (PDAC)

PDAC is the most common highly aggressive and lethal form of pancreatic cancer [130]. Li et al. [104] found that HDAC9 was highly expressed in primary PDAC tissue and PDAC cell lines (e.g., CFPAC-1, HPAC, SW1990 and Capan-2). HDAC9 expression in PDAC tissues was found to be negatively associated with tumor size and with T and N stages. PDAC patients with high levels of HDAC9 expression typically experience shorter periods of recurrence-free and disease-specific survival, which suggests that HDAC9 could be used as a prognostic predictor of PDAC. *In vitro* studies have revealed that HDAC9 promotes PDAC cell proliferation and migration.

### Oral squamous cell carcinoma (OSCC)

Bhawna et al. [105] suggested that HDAC9 may play a role in the pathogenesis OSCC. They found increased levels of HDAC9 (both mRNA and protein) in tissue samples from patients diagnosed with OSCC and in the human UPCI-SCC-116 OSCC cell line. OSCC patients with high levels of HDAC9 in their tumor tissue experienced significantly shorter periods of overall survival than patients whose tumor tissues had comparatively lower levels. In OSCC cells, HDAC9 may regulate transcription factor MEF2D and pro-apoptotic factor nuclear receptor subfamily 4 group A member 1 (NR4A1) to promote cell proliferation and decrease apoptosis [131]. This finding was reinforced in another study by Rastogi et al. [35], who found that miR-377 functions as a tumor suppressor and downregulates the expression of HDAC9 via direct interactions with its 3’ untranslated region, thereby resulting in an increased expression of NR4A1.

### Cutaneous squamous cell carcinoma (CSCC)

Fleming et al. [106] performed quantitative genotyping of single nucleotide polymorphisms (SNPs) and mapped candidate genes at a human skin tumor susceptibility locus in paired samples of both normal and tumor DNAs isolated from CSCC tissue. HDAC9 was identified as a candidate gene associated with CSCC because there was a preferential allelic imbalance of nine HDAC9 SNPs in CSSC tumors. Overall, the results from Fleming et al. [106] suggest that specific HDAC9 variants may be selected for during CSCC tumorigenesis. Additional functional, genetic and population-based studies will be needed to clarify the potential role of HDAC9 variants in CSCC initiation and/or progression.

###  Leiomyosarcoma (LMS)

LMSs are rare but highly malignant mesenchymal tumors derived from smooth muscle lineage cells [132]. HDAC9 is overexpressed in tumor tissue in about 30 % of LMS patients, and HDAC9 levels are inversely correlated with the overall LMS survival rate [107, 108]. Di Giorgio et al. [108] found that both HDAC9 and MEF2 were highly expressed in aggressive LMS cells. Eliminating the interactions between MEF2 and the HDAC9 promoter led to a significant decrease in LMS cell survival. Di Giorgio et al. [109] also demonstrated that LMS cells (SK-UT-1, SK-LMS-1, MES-SA and DMR) express HDAC9. In these cells, HDAC9 influenced the organization of the actin cytoskeleton and cell motility and downregulated the expression of the death receptor FasR. These results suggest that HDAC9 promotes LMS cell survival by repressing FasR expression.

###  Osteosarcoma (OS)

Osteosarcoma (also known as osteogenic sarcoma) is a common bone tumor that occurs predominantly in teenagers and young adults. HDAC9 is upregulated in osteosarcoma cell lines (e.g., U2OS and MG63) and in osteosarcoma tissues. Overexpression of HDAC9 in osteosarcoma cells promotes cell proliferation and invasion. Zhao et al. [110] studied the role of HDAC9 in the progression of osteosarcoma and found that HDAC9 binds to the proximal promoter region of the p53 gene and suppresses p53 transcription by regulating histone acetylation.

### Childhood acute lymphoblastic leukemia (ALL)

ALL is the most common childhood cancer and is the primary cause of cancer-related pediatric mortality. Moreno et al. [111] studied HDAC9 mRNA expression in bone marrow samples from children diagnosed with ALL and found that HDAC9 levels were higher in patients diagnosed with B-lineage CD10‐positive ALL. Multivariate analysis using the Cox regression model revealed that higher levels of HDAC9 expression were associated with a poor prognosis and a higher risk of unfavorable events. Notably, HDAC9 expression levels above the median value were associated with a reduced five-year event-free survival, especially in B-lineage CD10-positive ALL patients. The relationship between a poor prognosis and high levels of HDAC9 expression in B-cell precursor ALL patients was confirmed by Vega-García et al. [112], who analyzed mRNA expression levels in cells from pediatric patients diagnosed with acute leukemia.

### Acute myeloid leukemia (AML)

AML is an aggressive cancer that mostly arises in stem cells in the bone marrow of affected adults. High levels of HDAC9 expression in AML, as with other cancers, are significantly correlated with a diminished overall survival [112]. Bradbury et al. [113] explored HDAC9 expression in primary AML blasts and compared their results to HDAC9 levels in four other cell types, including quiescent or cycling CD34^+^ progenitor cells from umbilical cord blood and cycling CD34^+^ progenitors taken from peripheral mononuclear cells that were harvested from granulocyte colony-stimulating factor (GCSF)-stimulated adult donors. No significant differences in HDAC9 expression were observed among these cell types. Although HDAC9 expression was strongly and selectively induced in human AML HL60 cells exposed to the histone deacetylase inhibitor (HDI) sodium valproate in tissue culture, this response was not observed in the human KG1 AML cell line, in AML blasts, or in response to treatment with other HDIs. Further exploration of changes in HDAC9 expression in AML, particularly in response to the administration of one or more HDIs, will be important for fully understanding clinical responses and drug resistance [113].

### B-cell non-Hodgkin lymphoma (B-NHL)

HDAC9 is overexpressed in various lymphomas including B-NHL cell lines and primary B-NHL patient samples [15]. The HDAC9 locus at chromosome 7p21.1 is frequently amplified in B-NHL [114]. Gil et al. [115] generated transgenic mice that constitutively expressed HDAC9 during B cell development. These mice developed B-cell lymphoproliferative disorders with progression towards B-NHL, suggesting that HDAC9 expression is linked to lymphoid neoplasia. Gil et al. [115] also found that HDAC9 promoted cell growth and survival and modulated the activity of B-cell lymphoma 6 (BCL6) and p53 in B-NHL cells [115]. Dysregulated BCL6 activity may contribute to the development of B-cell lymphoma [133].

### Gastric cancer (GC)

GC, also known as gastric adenocarcinoma, is a malignant epithelial tumor. Xiong et al. [116] reported upregulated HDAC9 expression in human GC cell lines, including SGC-7901, BGC-823 and MKN-45. High levels of HDAC9 expression in primary tumor tissues from GC patients correlated with a lower survival rate. *In vitro* and *in vivo* experiments have both suggested a pro-oncogenic role for HDAC9 in GC, as HDAC9 knockdown resulted in reduced GC tumor growth and the induction of GC cell apoptosis and proliferation. Moreover, Xu et al. [31] found that miR-383-5p is a post-transcriptional inhibitor of HDAC9 expression, which suppresses GC progression by inhibiting its growth and inducing GC cell apoptosis.

### Lung cancer (LC)

Lung cancer is one of the most commonly diagnosed cancers worldwide. An integrative analysis of 60 paired lung adenocarcinoma specimens using a genome-wide human SNP array revealed that the aberrant *HDAC9* SNP rs10248565 on chromosome 7p21.1 may serve as a biomarker for lung adenocarcinoma in non-smoking women [118]. Okudela et al. [134] analyzed the expression of immunoreactive HDAC9 in surgically resected primary lung cancers. HDAC9 expression was lower in lung cancer cells than in non-tumor epithelial cells and was significantly lower in adenocarcinomas. Exogenous expression of HDAC9 reduced clonogenicity and proliferation in the immortalized airway epithelial NHBE-T and NSCLC (non-small cell lung cancer) cell lines A549 and H2087, respectively. These results suggest that HDAC9 may function as a tumor suppressor, most notably in lung adenocarcinomas. However, another study found that HDAC9 expression was upregulated in NSCLC. In an analysis of 337 tumor samples from patients diagnosed with NSCLC, Ma et al. [117] found that high levels of HDAC9 expression were correlated with reduced overall survival rates and poor clinical prognoses.

HDAC9 plays an important role in promoting growth and reducing apoptosis of NSCLC cells in studies performed both *in vivo* and *in vitro*. Guan et al. [135] investigated the involvement of a lncRNA known as CBR3 antisense RNA 1 (CBR3AS1) in the development of NSCLC. LncRNAs frequently act as inhibitory regulators of miRNAs by sequestering them or by competing with miRNAs for specific binding sites on mRNAs. CBR3AS1 is highly expressed in NSCLC tissues and cell lines and exhibits a pro-tumorigenic effect. A study focused on the underlying mechanism found that CBR3AS1 competed with endogenous miR5093p, which has been characterized as a tumor suppressor in NSCLC cells. HDAC9 is a direct target of miR5093p. It is, therefore, likely that the CBR3AS1/miR5093p/HDAC9 pathway plays an important role in the development and progression of NSCLC [34].

### Esophageal squamous cell carcinoma (ESCC)

ESSC ranks among the most common and deadly malignant tumors worldwide. Wang et al. [33] evaluated the molecular mechanisms underlying the actions of lncRNA LOC440173 in ESCC using both *in vitro* and *in vivo* tumor xenograft approaches. They showed that the LOC440173/miR-30d-5p/HDAC9 regulatory network promoted ESCC tumorigenesis. High levels of LOC440173 and HDAC9 have been reported in ESCC tissues and esophageal carcinoma cells. miR-30d-5p, which is downregulated in ESCC tissues, directly regulates HDAC9 expression.

### Bladder cancer (BCa)

BCa is a malignant tumor with a high incidence and recurrence rate. Lucca et al. [119] performed a gene array-based analysis to evaluate the expression of HDAC9 in RNA samples obtained from voided urine from patients with asymptomatic microscopic hematuria who were diagnosed with urothelial BCa. They found that HDAC9 mRNA was upregulated in urothelial BCa patients. Moreover, Wang et al. [32] found that HDAC9 was upregulated in BCa tumor tissues and cell lines (including 5637 and T24). HDAC9 expression was suppressed by miR-211-5p. In *in vitro* cell experiments, overexpression of miR-211-5p or downregulation of HDAC9 significantly inhibited BCa cell proliferation and migration and induced their apoptosis. Overall, the results from Wang et al. [32] suggest that HDAC9 may function as an oncogene to promote BCa development.

### Retinoblastoma (Rb)

Rb is the most frequent malignant intra-ocular tumor in children. HDAC9 is expressed in Rb tissues, and HDAC9 overexpression has been associated with a poor prognosis in Rb patients. HDAC9 expression levels were positively associated with the proliferation of human Rb cells, including Y79 and WERI-Rb-1. Zhang et al. [120] found that downregulation of HDAC9 induced cell cycle arrest at the G1 phase with significant decreases in cyclin E2 and CDK2 expression *in vitro*. HDAC9 also inhibited Rb tumor growth in a mouse xenograft model. The pro-oncogenic effects of HDAC9 in Rb were further evaluated by Xu et al. [29], who found that HDAC9 is a direct target of miR-936. In these experiments, HDAC9 counteracted the tumor‑suppressive actions of miR‑936 in Rb cells. In a similar study, Jin et al. [30] demonstrated that the tumor suppressor miR-101-3p directly downregulates HDAC9 expression and thereby suppresses Rb cell proliferation.

### Brahma-negative carcinoma

Brahma (BRM) is an anti-oncogene that binds to the retinoblastoma protein (RB) to facilitate its function [136]. BRM frequently undergoes epigenetic silencing in a variety of tumor types, and loss of BRM can potentially inactivate RB-associated pathways [137]. Kahali et al. [121] found that HDAC9 can regulate the expression of BRM. HDAC9 is highly expressed in BRM-negative carcinoma cell lines including SW13 and C33A, which are derived from adrenal and cervical carcinomas, respectively. Specific knockdown of HDAC9 in BRM-negative carcinoma cells led to an induction of BRM expression and a significant suppression of cell growth. HDAC9 overexpression, therefore, appears to be relatively specific for BRM-negative tumor cells.

**Fig. 3:**
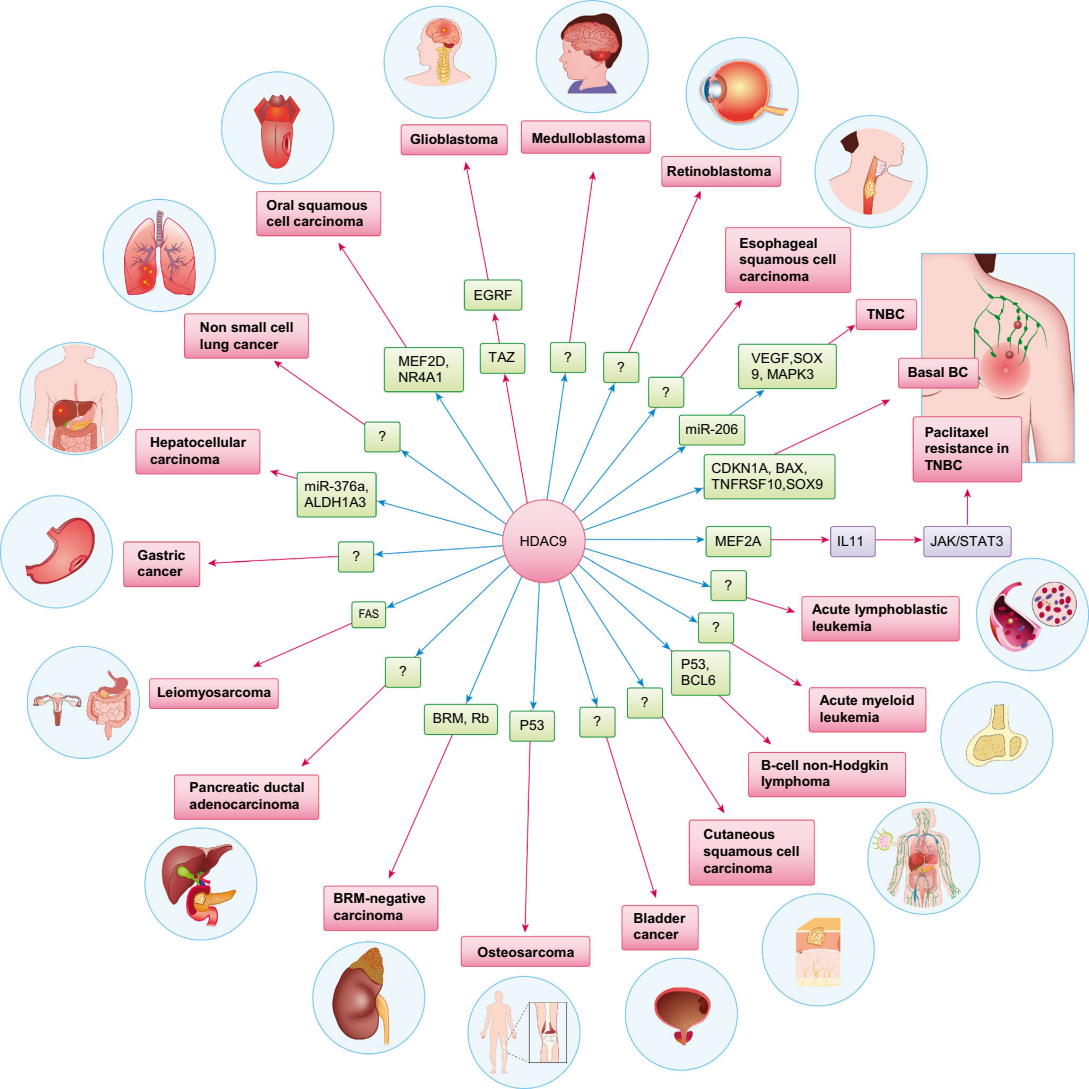
Potential targets and interacting proteins of HDAC9 involved in the specific types of cancer. TNBC: triple negative breast cancer

Overall, dysregulated HDAC9 expression has been linked to a variety of cancers (Fig.  3). Because HDAC9 expression is generally correlated with a poor prognosis and reduced survival rates, the detection of HDAC9 in cancer patients may be of prognostic value. Although the findings reviewed in this section broadly suggest that HDAC9 has pro-oncogenic activity, they also show that HDAC9 may behave differently depending on the tissue context and tumor type. For example, Okudela et al. [138] reported that HDAC9 may function as a tumor suppressor in lung adenocarcinomas. HDAC9 can also deacetylate ataxia-telangiectasia group D-complementing protein (ATDC), thereby altering its interactions with p53 and the expression of p53-regulated genes [139, 140]. HDAC9 may also acts as a tumor suppressor in pancreatic cancer, even though this activity has not been specifically demonstrated.

Despite this general understanding of the relationship between HDAC9 and cancer development/progression, the specific role(s) played by HDAC9 in most types of cancer, as well as the mechanisms underlying its aberrant expression and the associated functional consequences, are as yet not fully understood. Future studies should, therefore, focus on gaining deeper insight into the mechanisms underlying HDAC9-associated activities in individual types of cancer.

## HDAC9 inhibition in cancer therapy

Because high levels of HDAC9 are frequently associated with advanced malignancy and poor clinical outcomes, therapeutics that target HDAC9 might be explored for the treatment of one or more types of cancer. Although many HDAC inhibitors have shown strong anti-tumor efficacies in preclinical studies, and four have already been approved by the United States Food and Drug Administration (FDA), all currently existing HDAC inhibitors have been found to be associated with side effects linked to their concomitant inhibitory activities against multiple HDACs [141, 142]. There is, therefore, an urgent need for the development of selective HDAC inhibitors that exhibit less side effects while maintaining anti-tumor activity [10]. The development of selective HDAC9 inhibitors has been particularly challenging due to the high degree of sequence homology exhibited by zinc-dependent HDACs. Boskovic et al. [143] identified a novel, hydroxyquinoline-containing compound, BRD4354, that is capable of a preferential reversible and time-dependent inhibition of HDAC5 and HDAC9. However, there are currently no reports detailing the *in vivo* inhibitory efficacy of this compound, and no natural substrates of class IIa HDACs, including HDAC9, have been identified yet [144].

## Conclusions and clinical perspectives

HDAC9 expression is tissue- and cell type-specific. This enzyme functions as part of a protein complex that recruits transcription factors to regulate the acetylation of target regulatory proteins. Recent studies that have used expression profiling and examined epigenetic alterations suggest that HDAC9 plays a crucial role in the development of pathology, especially in the development and progression of cancer. Although HDAC9 has recently received increased attention, many important questions have not yet been fully addressed. For example, it is not yet clear which transcription factors interact with HDAC9 and how they regulate its expression in specific biological contexts. Although some of the genes controlled by HDAC9 have been identified, the precise mechanisms associated with HDAC9 and the corresponding network of interactions remain to be defined. The factors that regulate HDAC9 function during tumorigenesis also remain largely unknown. Future studies should focus on clarifying the role of HDAC9, identifying its targets and functional partners, and determining which transcription factors regulate HDAC9 expression in each circumstance. In-depth knowledge of the role of individual HDAC9 isoforms in promoting oncogenic pathways and malignant responses will also facilitate the development of selective HDAC9 inhibitors. Efforts to target the most relevant HDAC9 isoform for each clinical indication might be an empirical way to improve drug efficacy and reduce the number of side effects that result from the concomitant inhibition of multiple isoforms.

Because differential expression of HDAC9 has been found to be associated with a poor prognosis and reduced survival rates for several types of cancer, detection of HDAC9 and its interacting partners may be of important prognostic value. Advanced techniques (e.g., high throughput analyses, mRNA or proteins array systems, targeted mass spectrometry) could provide a more differentiated evaluation of the expression profiles of HDAC9 and its partners within different tissues and cancer types. These approaches, together with molecular and clinical studies, have the potential to identify biomarkers for the accurate and sensitive detection of certain types of cancer in clinical samples and for prognoses, risk stratifications and personalized treatment decisions.

Although the development of selective HDAC9 inhibitors has proven challenging, there are a few approaches that may specifically inhibit HDAC9 expression. For example, HDAC9 can be inhibited using RNA interference (RNAi)-based approaches through target-specific HDAC9 mRNA degradation. Single-stranded microRNAs (miRNAs) and double-stranded small interfering RNAs (siRNAs) are integral to RNAi-based therapy. In some types of cancer, HDAC9 expression is regulated by specific miRNAs, so delivering these particular miRNAs or miRNA inhibitors into cancer cells may restore the normal protein level of HDAC9. In addition, siRNAs can be designed to inhibit specific HDAC9 isoforms, which ensures that the therapeutic approach targets the most relevant isoform in a specific indication and eliminates the toxicities associated with the inhibition of multiple isoforms.

It is now widely recognized that therapeutic approaches that combine two or more agents can result in a synergistic effect through different mechanisms of action [145]. For example, HDAC9 inhibitors administered in combination with conventional anti-tumor agents or novel target-specific agents (that target known cancer-associated pathways for each type of cancer) may increase therapeutic efficacies. However, most RNA-based therapeutics are unstable and require a delivery system, and so various delivery vehicles have been developed to improve the transport and bioavailability of oligonucleotides [138]. One of the most widely used delivery systems are lipid nanoparticles (LNPs) which can encapsulate negatively charged oligonucleotides and reach target cells. Most interestingly, LNPs can encapsulate both RNAi molecules and conventional drug compounds to reduce resistance or improve efficiency. We have shown that liposome switchable LNPs co-delivering miR-181a and melphalan significantly increased the therapeutic impact on retinoblastoma [146]. To improve the selectivity toward target cells and to reduce the amount of drugs reaching non-target tissues, delivery vehicles can be decorated with active targeting ligands like folic acid, hyaluronan, PSMA (prostate-specific membrane antigen), and their pairing receptors folate receptor, CD44 and PSMA receptor, which are overexpressed mainly in tumor cells [147]. Nanoparticles coated with these ligands have shown specific and efficient delivery of therapeutic agents into cancer cells in several preclinical studies [148–150]. Given our in-depth knowledge of the roles of HDAC9, the development of targeted delivery nanoparticles for combinational treatment (HDAC9 targeted therapy with traditional therapeutics) may be a viable approach for future clinical applications.

## Data Availability

Not applicable.

## Abbreviations

ABCA1: ATP-binding cassette transporter A1
ABCG1: ATP-binding cassette transporter G1
AChR: acetylcholine receptor
AES: amino enhancer of split
AL: acute lymphoblastic leukemia
AML: acute myeloid leukemia
ATDC: ataxia telangiectasia group D-complementing protein
BC: breast cancer
BCa: bladder cancer
BCL6: B-cell lymphoma 6 protein
BMMSCs: bone marrow mesenchymal stem cells
C/EBPα: CCAAT/enhancer binding protein-α
CaMK: calcium calmodulin-dependent protein kinase
CRM1: chromosomal region maintenance protein 1
CtBP: C-terminal-binding protein
DCs: dendritic cells
Dnmt3a: DNA methyltransferase 3A
EGFR: epidermal growth factor receptor
ER: estrogen receptor
ESCC: esophageal squamous cell carcinoma
FABP4: fatty acid-binding protein 4
FOXO1: forkhead box protein O 1
FOXP3: forkhead box P3
GATA3: GATA binding protein 3
GC: gastric cancer
GLI1: glioma-associated oncogene homolog 1
HCC: hepatocellular carcinoma
HCV: hepatitis C virus
HDAC: histone deacetylase
HDI: histone deacetylase inhibitor
HFD: high fat diet
IL11: interleukin-11
IFN: interferon
IRF3: interferon regulatory factor 3
KO: knockout
LMS: leiomyosarcoma
lncRNA: long noncoding RNA
MAPK3: mitogen-activated protein kinase 3
MB: medulloblastoma
MEF2: myocyte enhancer factor 2
MEF2: myocyte-specific enhancer binding factor-2 D
MEG3: maternally expressed gene 3
MITR: MEF2-interacting transcriptional repressor
MRL/lpr: Murphy Roths large/lymphoproliferation strain
MSCs: mesenchymal stem cells
N-CoR: nuclear receptor co-repressor
NES: nuclear export signal
Nkx3.2: NK3 homeobox 2
NLS: nuclear localization signal
NR4A1: nuclear receptor subfamily 4 group A member 1
NSCL: non‑small cell lung cancer
OSCC: oral squamous cell carcinoma
OXA: oxaliplatin
PIASy: protein inhibitor of activated STAT
PPARγ: peroxisome proliferator-activated receptor gamma
RANKL: receptor activator of nuclear factor κB ligand
Rb: retinoblastoma protein
RNF4: ring finger protein 4
Shh: sonic hedgehog
SIKs: salt inducible kinases
SNP: single nucleotide polymorphism
SOX9: SRY-box transcription factor 9
STAT5: signal transducer and activator of transcription 5
SUMO: small ubiquitin-like modifier
TAZ: transcriptional co-activator with PDZ-binding motif
TBK1: TANK-binding kinase 1
TCGA: The Cancer Genome Atlas
TFs: transcription factors
TNBC: triple-negative BC
Treg: regulatory T cell
USF1: upstream stimulatory factors 1
VEGF: vascular endothelial growth factor
